# Supplementary material for: The Generation of a H9N2 Avian Influenza Virus with HA and C3d-P29 Protein Fusions and Vaccine Development Applications
Source: Vaccines (Basel). 2025 Jan 21;13(2):99. doi: 10.3390/vaccines13020099 (PMC11861068; doi:10.3390/vaccines13020099)
Supplement: Supplementary file 1 [file vaccines-13-00099-s001.zip › vaccines-3409111-supplementary.pdf]

Table 1. The nucleotides of different linkers.

Linker and P29	Sequence (5' → 3')
(GGGGS)-1	GGTGGCGGAGGGAGT
(GGGGS)-2	GGCGGGGGAGGTAGC
(GGGGS)-3	GGAGGTGGCGGGTCT
(GGGGS)-4	GGGGGCGGTGGATCC
P29	AAAGTCCTGATGAAGTTCTCCAAAGATGGCACCCACTGGGCGGAA CGCAACGCCCACACCTACAACATCGAGGGGACGTCCTACGCT

Table 2. The primers for PCR and RT-PCR.

Primer name	Sequence (5' → 3')
HA1-F	ATGGAGACAGTATCA
HA1-R	ACTCCCTCCGCCACCTGCATAGCTTACTGTTG
HA2-F	GGGGGCGGTGGATCCGATAAAATCTGCATCGGCTACCAATC
HA2-R	CTATATACAAATGTTGCATC
P29.1-F	GGTGGCGGAGGGAGT
P29.1-R	GCTACCTCCCCCGCC
P29.2-R	AGACCCGCCACCTCC
P29.3-R	GGATCCACCGCCCCC
Pcaggs-HA-P29.N-F	GTCTCATCATTTTGGCAAAG ATGGAGACAGTATCA
Pcaggs-HA-P29.N-R	AGGGAAAAAGATCTGCTAGC CTATATACAAATGTTGCATC
PHW-HA-P29.N-F	GGGGAGCAAAAGCAGGGGATA
PHW-HA-P29.N-R	GGTTATTAGTAGAAACAAGGGTGTTTT
PHW-PB2-F	CCAGCGAAAGCAGGTC
PHW-PB2-R	TTAGTAGAAACAAGGTCGTTT
PHW-PB1-F	CACACAGCTCTTCGGCCAGCGAAAGCAGGCA
PHW-PB1-R	CACACAGCTCTTCTATTAGTAGAAACAAGGCATTT
PHW-PA-F	CCAGCGAAAGCAGGTAC
PHW-PA-R	TTAGTAGAAACAAGGTACTT
PHW-HA-F	TTAGTAGAAACAAGGGTGTTTT
PHW-HA-R	CCAGCAAAAGCAGGGG
PHW-NP-F	CACACAGCTCTTCGGCCAGCAAAAGCAGGGTA
PHW-NP-R	CACACAGCTCTTCTATTAGTAGAAACAAGGGTATTTTT
PHW-NA-F	CACACAGCTCTTCGGCCAGCAAAAGCAGGAGT
PHW-NA-R	CACACAGCTCTTCTATTAGTAGAAACAAGGAGTTTTTT
PHW-M-F	CACACAGCTCTTCTATTAGCAAAAGCAGGTAG
PHW-M-R	CACACAGCTCTTCGGCCAGTAGAAACAAGGTAGTTTTT
PHW-NS-F	CACACAGCTCTTCTATTAGCAAAAGCAGGGTG
PHW-NS-R	CACACAGCTCTTCGGCCAGTAGAAACAAGGGTGTTTT
chIFN-α-F	CCTTCCTCCAAGACAACGATTAC
chIFN-α-Probe	TTGTGGATGTGCAGGAACCAGGC
chIFN-α-R	AGTGCGAGTGATAAATGTGAGG
chIFN-β-F	CCTTGAGCAATGCTTCGTAAAC
chIFN-β-Probe	CAACGCTCACCTCAGCATCAACAA
chIFN-β-R	GGAAGTTGTGGATGGATCTGAA
chIFN-γ-F	GTGAAGAAGGTGAAAGATATCATGGA

chIFN-γ-Probe	TGGCCAAGCTCCCGATGAACGA
chIFN-γ-R	GCTTTGCGCTGGATTCTCA

Table 3. The information of the rH514 and rH514-P29.N (N=1, 2) viruses.

**Virus**	**HA titer (Log_2)**	**EID_{50} (Log_{10}/mL)**
rH514	10	9.50
rH514-P29.1	10	9.50
rH514-P29.2	9	9.50

The original western blot figures

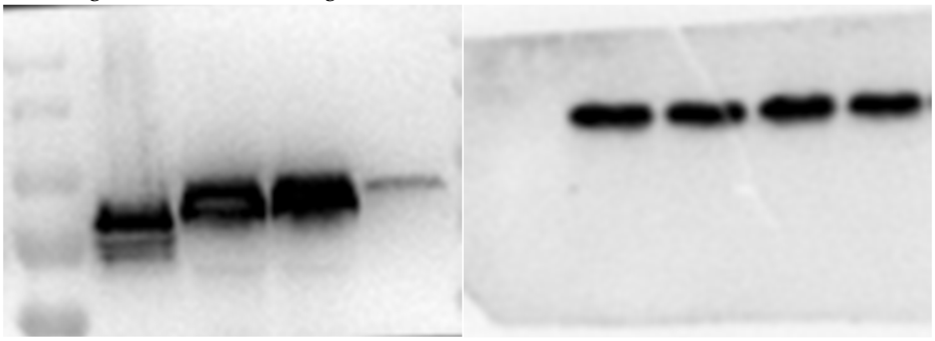

Figure 1C

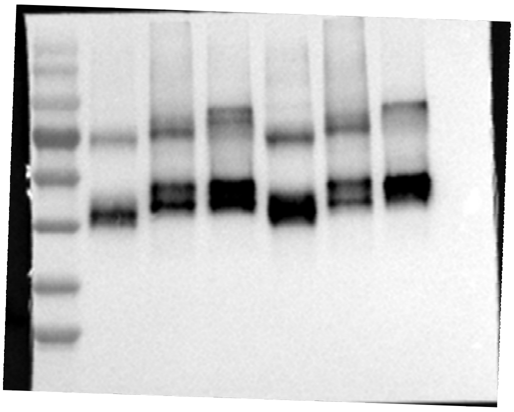

Figure 3B
